# Literary Notes

**Published:** 1905-02

**Authors:** 


### LITERARY NOTES.

Messrs. William Wood & Company, New York, have issued a brochure entitled, One hundred years of publishing (1804-1904). It is handsomely printed on heavy calendered paper, is well illustrated with portraits of the several members of the firm from its establishment down to the present time, and pictures of the different buildings it has occupied during the last century. It contains an interesting history, covering a lengthened period of time, during which nearly all of the best printing in the world has been evolved.

---

The *Saint Louis Courier of Medicine* is publishing a series of articles on the Care of premature infants written by Dr. Zahorsky, in which he gives his full experience relating to the care of premature infants and the baby incubator at the Louisiana Purchase Exposition.

